# A Wolf in Sheep's Clothing: Extensive Musculoskeletal and Cutaneous TB Masquerading as Primary Erythema Nodosum

**DOI:** 10.1155/crdi/5905265

**Published:** 2025-10-25

**Authors:** Tanner Shull, Hemil Gonzalez

**Affiliations:** ^1^Rush Medical College, Rush University Medical Center, Chicago 60608, Illinois, USA; ^2^Division of Infectious Diseases and Internal Medicine, University of Texas Medical Branch, Galveston 77555, Texas, USA; ^3^Moody Brain Health Institute, University of Texas Medical Branch, Galveston 77555, Texas, USA

**Keywords:** musculoskeletal, osteomyelitis, renal transplant, septic arthritis, septic tendinitis, solid organ transplant, tuberculosis

## Abstract

**Introduction:**

Tuberculosis (TB) causes substantial morbidity and mortality globally, with continued endemicity in developing populations. Most cases of primary TB present as pulmonary TB; however, between 10% and 20% include extrapulmonary manifestations. Almost 26% of extrapulmonary manifestations include musculoskeletal TB. The risk of reactivation of latent TB is approximately 10% per year and is most common in those with immunocompromising conditions. Diagnosis of extrapulmonary TB remains elusive because of atypical presentation.

**Case:**

We present a case of a 71-year-old man with a history of renal transplantation and previously treated latent TB who presented with worsening left lower extremity pain and swelling over the previous six months. Initially, the patient was unsuccessfully treated for presumed bacterial cellulitis with several rounds of antibiotics. The patient was then seen by Dermatology, who diagnosed presumed primary erythema nodosum. He was managed with prednisone and topical steroids for several months with only partial improvement. At admission, physical exam was significant for a 4 × 3-cm erythematous, warm, and tender area on the lower left extremity. Labs showed elevated neutrophils and lymphopenia, and an MRI of the limb suggested hindfoot abscesses, talonavicular septic arthritis, and surrounding osteomyelitis. Intraoperative cultures grew acid-fast bacilli and were confirmed to be pan-sensitive *M. tuberculosis* complex.

**Conclusion:**

Musculoskeletal TB diagnosis requires a high index of suspicion, thorough physical exam, imaging, and tissue for culture and biopsy. Special attention should be placed on the combined risk factors of previous TB diagnosis, immunocompromised status, and symptoms refractory to alternative management strategies such as antibiotics and corticosteroids.

**Summary**

• Premature closure on left lower extremity erythema nodosum as a primary diagnosis from targeted limb evaluation.
• Anchoring on the diagnosis of erythema nodosum once lymphangitic spread began to develop.
• Immunocompromised patient with a high risk of extrapulmonary TB (EPTB) given history of/prior renal transplant and previous history of latent pulmonary TB.

## 1. Introduction

Tuberculosis (TB) is the most substantial infectious killer from a single pathogen globally [1]. An estimated 1.7 billion people worldwide are latently infected with TB (LTBI) [2]. Among those without immunocompromised conditions, progression to primary TB remains below 10% [3–5]. In 2023, approximately 10.8 million people globally had primary TB, with 1.3 million deaths [1].

Most primary TB cases reported refer to pulmonary manifestations. However, between 10% and 20% of all primary TB may include extrapulmonary manifestations [6–8]. Extrapulmonary TB (EPTB) includes TB manifestations in pleural, lymphatic, musculoskeletal, and other body compartments not associated with the lungs [9]. EPTB prevalence is often underestimated, given underreporting in areas of high incidence [10, 11], limited sampling of extrapulmonary sites [12, 13], and frequent concomitant involvement that is reported as pulmonary TB only [14, 15]. Furthermore, microbiological tests for pulmonary TB, such as PCR and sputum culture, are less predictive of EPTB involvement. In EPTB, samples from debridement may not always be available [16, 17].

We present a case highlighting the importance of a comprehensive evaluation and high index of suspicion of reactivated LTBI in the context of an immunocompromised host.

## 2. Case

**Figure 1:**
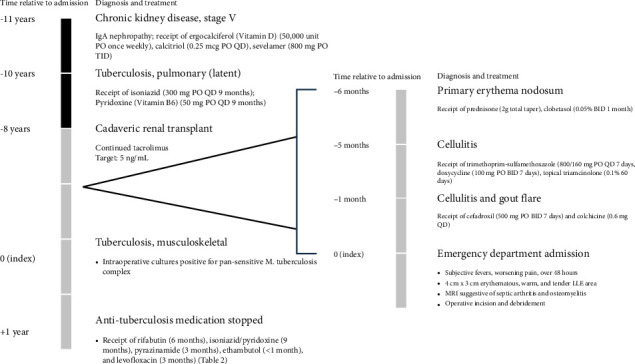
Chronological patient history. Abbreviated timeline showing significant past medical and surgical history of the patient. Associated diagnoses, medications, and clinical notes are included. Abbreviations: LLE: lower left extremity; MRI: magnetic resonance imaging.

A 71-year-old man originally from a TB-endemic country presents with worsening lower extremity swelling and redness over the previous six months (Figure 1). His past medical history is significant for cadaveric renal transplant 7 years prior. Prior to CRT, he was found to have LTBI and received a 9-month INH/B6 regimen. Upon completion of therapy, his chest X-ray remained unchanged, and he proceeded with CRT. Immediately following CRT, antirejection medications included alemtuzumab (Campath), tacrolimus, mycophenolate, and prednisone. Since CRT, he has maintained serum tacrolimus levels of 5 ng/mL.

**Figure 2:**
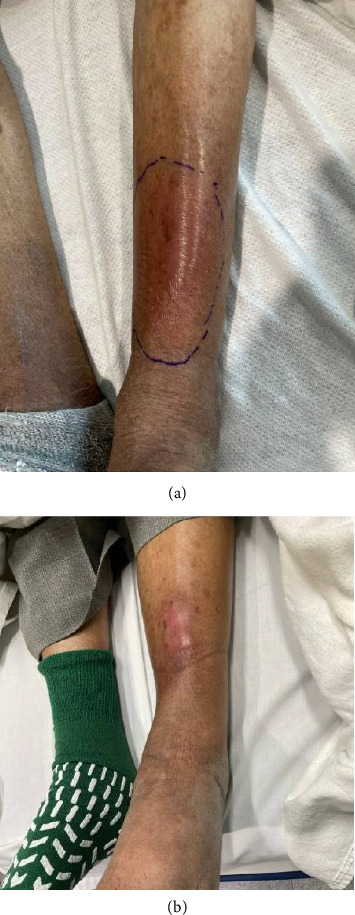
Clinical media showing lower left extremity redness and swelling at two different timepoints. (a) Lower left extremity redness and swelling 6 months before index admission. (b) Index admission with 4 cm × 3-cm focal area of warm, tender, indurated left extremity redness, and swelling.

Six months before index admission, he presented to clinic with “ill-defined erythematous plaques to the left lateral leg” (Figure 2(a)), which was diagnosed as primary erythema nodosum. He was treated with tapered oral prednisone and topical clobetasol.

The following month, he experienced several episodes of swelling and redness of the limb. He was started on trimethoprim-sulfamethoxazole for cellulitis. His symptoms persisted, however, which prompted a medication switch to doxycycline and topical triamcinolone.

His lesions continued to progress for the next 3 months. He received cefadroxil and colchicine for a suspected gout flare compounded with cellulitis despite neither history of gout nor altered biomarkers. Notably, the patient endorsed improved redness and swelling, but no improvement in pain.

**Figure 3:**
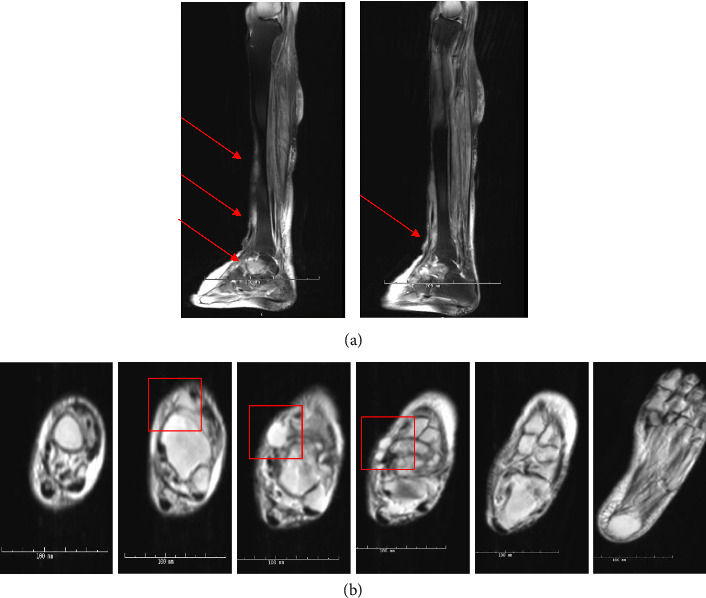
Magnetic resonance imaging of the lower left limb at index admission. (a) Sagittal evaluation of the lower left limb with associated high density opaque regions (red arrows). (b) Evaluation of the lower left limb at index admission across caudal axial series with associated high density opaque regions (red arrows).

The patient presented to the emergency department one month later with subjective fevers of 101^o^F and worsening left lower extremity swelling, redness, and pain for the previous three days. On exam, the patient was ill-appearing but in no acute distress, with pitting edema to shins bilaterally with a 4 × 3-cm area of warmth, induration, tenderness, and erythema over the anterior shin of the lower left extremity (Figure 2(b)). Initial labs showed elevated inflammation markers (CRP and ESR). Blood count showed acute elevated neutrophils and lymphopenia. The patient was also chronically anemic and thrombocytopenic. An MRI was obtained, which demonstrated septic arthritis of the talonavicular joint, talus and navicular osteomyelitis, and infectious tenosynovitis of the posterior tibial tendon (Figure 3). The patient was scheduled for an operative incision and drainage of tissue.

In the operating room, gross purulence was found in the dorsal talonavicular joint capsule with significant joint synovitis. Upon blunt dissection and debridement, additional purulence was noted tracking to the hindfoot. An accessory incision was made with additional gross purulence along the posterior tibial tendon sheath, which appeared degenerated. A second operation was scheduled and performed, showing further gross purulence and completely lysed talonavicular joint cartilage from the subchondral bone. Samples from each section were obtained and sent for culture (Table 1).

Acid-fast bacilli (AFB) were identified by fluorochrome stain, and *M. tuberculosis* complex was identified by DNA probe assay. A total of six operating room samples were evaluated (Table 1). Three sputum samples were obtained, with one sample showing *M. tuberculosis* complex by DNA probe assay, despite a negative fluorochrome stain. The other two samples were negative for *M. tuberculosis* complex following 8 weeks of incubation. All samples were found to be pan-susceptible *M. tuberculosis* complex (Table 2).

Anti-TB medication was started with rifabutin instead of rifampin to prevent drug interactions with tacrolimus. Approximately 1 week following initiation, liver function tests (LFTs) were consistently elevated for unclear reasons. The patient was found to have reactivated hepatitis B infection (VL > 6M copies/mL) and was started on entecavir. After 12 months of therapy, the HBV viral load was rechecked and reported as 153 copies/mL.

LFTs were continually monitored, and the patient completed 2 months of intensive RIPE therapy before transitioning to RIF/INH therapy (Table 2). Given pan-susceptibility, ethambutol therapy was discontinued after 1 month. The patient experienced a lapse in treatment during Month 6 because of acute thrombocytopenia. Approximately 1 month later, he was restarted on RIF/INH but quickly discontinued because of concern for rifabutin allergy. The patient finished the remaining 3 months of anti-TB therapy on INH and levofloxacin (Table 2).

Approximately 1 year after treatment initiation and upon receipt of 9 months of anti-TB therapy, the patient's anti-TB medications were stopped. He continues to do well without any relapse.

## 3. Discussion

We present a case of a patient born in a TB-endemic country with a past medical history significant for CRT and LTBI currently receiving immunosuppressive therapy. He had been experiencing chronic lymphocutaneous abnormalities in his lower extremities without significant systemic symptoms. Notably, his symptoms had neither responded to typical antibacterial therapies nor corticosteroids as he was being managed alternatively for bacterial as well as primary inflammatory processes. By index admission, he had systemic symptoms including fevers, malaise, and new onset pain localized to joints and other musculoskeletal structures of his lower extremities. Intraoperative cultures grew pan-susceptible *M. tuberculosis* complex.

This case highlights heuristic pitfalls physicians may experience when evaluating patients with clinically evident, albeit undiagnosed TB.

Prioritizing primary inflammatory causes versus infectious causes in our immunocompromised patient resulted in missed diagnostic evaluations for opportunistic infections. An erythematous, inflamed plaque in a transplant patient should be considered infectious until diagnostically proven otherwise. Transplant patients are a high-risk group due to antirejection therapies. In antirejection therapies, lymphocytes are primary targets, which permit the reactivation of latent TB [18]. Tacrolimus specifically inhibits inflammatory processes involving IL-2 transcription, degranulation of immune cells, and T-cell proliferation [19]. Each predisposes LTBI to activation and secondary infection.

False reassurance due to prior therapy represents another heuristic error. Given its long duration of treatment and side effect profile, completion rates of LTBI therapy remain low [20]. Furthermore, this patient presented with a classic syndrome of erythema nodosum, which has been associated with TB [21, 22]. Traditionally, erythema nodosum presents as bilateral and symmetric multinodular foci. Despite our patient's atypical appearance of erythema nodosum, providers did not investigate secondary causes of erythema nodosum or obtain a skin biopsy, which incorrectly adjudicated symptoms to a primary inflammatory etiology rather than an infectious one. Glucocorticoids give the appearance of improvement even in infectious conditions by reducing inflammation. Deeper tissues such as tendons, joints, and bones were still affected in our patient. Despite worsening clinical presentation, providers continued to treat for primary erythema nodosum, until his index emergency department admission.

In conclusion, LTBI reactivation in immunosuppressed patients can be prevented with judicious use of additional immunosuppressive medications and establishing a comprehensive differential diagnostic evaluation for patients from TB-endemic regions with significant past medical and surgical history. Physicians can further suspect LTBI reactivation in worsening inflammatory and immune processes not responsive to other refractory anti-inflammatory and antibiotic therapies.

## Figures and Tables

**Table 1:** Clinical microbiology results.

Date	Culture AFB with smear	DNA probe assay	Fluorochrome stain
Hospital day 2	Sputum	Positive *M. tuberculosis* complex identified by DNA after 1 week	“No AFB seen fluorochrome stain”
Hospital day 2, operation 1	Talonavicular joint synovial fluid	Positive *M. tuberculosis* complex	“Many AFB seen fluorochrome stain”
Hospital day 2, operation 1	Deep foot abscess	Positive *M. tuberculosis* complex	“Many AFB seen fluorochrome stain”
Hospital day 2, operation 1	Left foot talonavicular joint synovium	Positive *M. tuberculosis* complex	“Moderate AFB seen fluorochrome stain”
Hospital day 3	Sputum	Negative	No AFB seen fluorochrome stain after 8 weeks
Hospital day 4	Sputum	Negative	No AFB seen fluorochrome stain after 8 weeks
Hospital day 6, operation 2	Left hindfoot abscess	Positive *M. tuberculosis* complex	“Many AFB seen fluorochrome stain”
Hospital day 6, operation 2	Navicular bone	Positive *M. tuberculosis* complex	“Many AFB seen fluorochrome stain”
Hospital day 6, operation 2	Talus bone	Positive *M. tuberculosis* complex	“Many AFB seen fluorochrome stain”

*Note:* Culture sources for acid-fast bacilli (AFB) with smear are shown with date of sample, accompanying DNA probe assay, and fluorochrome stain results.

**Table 2:** Antituberculosis medications and susceptibility.

**A: Susceptibility study for isolated *M. tuberculosis* complex**		
**Component and dose**	**Culture AFB with smear**	

Ethambutol 5.0 μg/mL	Susceptible	
Isoniazid 0.1 μg/mL	Susceptible	
Pyrazinamide 100 μg/mL	Susceptible	
Rifampin 1.0 μg/mL	Susceptible	

**B: Medication receipt for the patient is shown with component, accompanying dosing, months of duration, and considerations for our patient**		
**Component**	**Dosing**	**Considerations**

*Initiation phase (6 months)*		
Rifabutin	150 mg PO BID	Rifampin is a strong inducer of the CYP-450 family (CYP3A4). Tacrolimus levels would decrease in this patient, increasing risk of transplant rejection for long-term anti-TB therapy. Rifabutin is a weak inducer of CYP3A4 and therefore a better alternative for this patient.
Isoniazid	300 mg PO QD	Addition of pyridoxine to prevent neuropathy associated side effects.
Pyrazinamide	1000 mg PO QD	Discontinued following 3 months
Ethambutol	800 mg PO QD	Discontinued after 1 month, after *M. tuberculosis complex* was found to be pan-sensitive.

*Continuation phase (3 months)*		
Isoniazid	300 mg PO QD	
Levofloxacin	750 mg PO QD	Allergy to rifabutin was suspected following interruption of RIF/INH at 6 months.

## Data Availability

The data used and/or analyzed during this study are available from the corresponding author upon reasonable request.
